# University Students’ Antibiotic Use and Knowledge of Antimicrobial Resistance: What Are the Common Myths?

**DOI:** 10.3390/antibiotics9060349

**Published:** 2020-06-20

**Authors:** Nurul Shaheera Shahpawee, Li Ling Chaw, Siti Hanna Muharram, Hui Poh Goh, Zahid Hussain, Long Chiau Ming

**Affiliations:** 1 PAPRSB Institute of Health Sciences, Universiti Brunei Darussalam, BE 1410 Bandar Seri Begawan, Brunei; 16b3010@ubd.edu.bn (N.S.S.); liling.chaw@ubd.edu.bn (L.L.C.); hanna.muharram@ubd.edu.bn (S.H.M.); pohhui.goh@ubd.edu.bn (H.P.G.); 2 Faculty of Health, University of Canberra, Canberra 2617, Australia; Zahid.Hussain@canberra.edu.au

**Keywords:** cross-sectional survey, antibiotic use, antimicrobial resistance, knowledge, Brunei

## Abstract

We aimed to assess antibiotic usage and knowledge regarding antibiotics and antimicrobial resistance (AMR) among undergraduate students of the Universiti Brunei Darussalam (UBD), public university located in Brunei Darussalam. A cross-sectional study was performed using a self-administered questionnaire. The questionnaire was adapted from the World Health Organization’s (WHO) “Antibiotic resistance: Multi-country public awareness” survey distributed online. Students at the UBD were invited to participate in the online survey through internal email. The questionnaire consisted of five sections: demographic information, antibiotic usage, knowledge on antibiotics, antibiotic resistance (AMR), and use of antibiotics in agriculture. The data were analyzed descriptively and appropriate inferential statistics were used accordingly. A total of 130 students returned a completed questionnaire. The result of the study found that 51% (n = 66) of the students had good level of knowledge of antibiotic and antimicrobial resistance with a mean total knowledge score of nine out of 14. Of note, 76% (n = 99) of the respondents mistakenly believed that antibiotic resistance is the result of the body becoming resistant to antibiotics. Only 14% (n = 18) of the respondents were found to have poor knowledge on antibiotics and antimicrobial resistance in the study. Misconceptions in regards to the use of antibiotics for conditions related to viral illnesses like cold and flu (41%, n = 53) were noticed among the respondents in our study. Thus, improving knowledge on antibiotics is crucial to address these beliefs.

## 1. Introduction

The abuse and excessive utilization of antibiotics have led to the rise of antimicrobial resistance (AMR) which poses a major threat to individual health globally [1]. At least 700,000 deaths globally a year are caused by drug-resistant diseases and the rate of mortality has been predicted to grow to 10 million deaths per year by 2050 [2,3]. In Asia alone, an estimated 4.73 million deaths annually will be attributed to antimicrobial resistance by 2050 [4].

Antimicrobial drugs can be defined as a group of drugs that are effective against microorganisms and comprise antibiotics, antifungals, antivirals, and antiparasitic drugs, whereas an antibacterial agent is any agent that is directed against bacteria [5]. Bacterial infections can be prevented and treated with medicines called antibiotics [6]. An antibacterial agent only acts upon bacteria, but antibiotics can work on both bacteria and fungi.

AMR occurs when microorganisms such as bacteria develop resistance to an antimicrobial agent. Due to the emergence of resistance, the ability to treat various infectious diseases becomes less effective thus causing treatment failure, increasing health care cost, and even leading to death [7]. The factors causing the growth of resistance include limited knowledge and awareness of antibiotics, as well as inadequate education regarding antibiotic usage of the prescriber [8]. Inappropriate use, such as incomplete antibiotic treatment, reuse of leftover medicines, mistakenly taking antibiotics for the treatment of viral infections, self-medication, and skipping of doses, has been reported [9]. Indeed, many studies have implied that patients sometimes do not fully comply with the antibiotic regimen [10,11,12]; this may result in the failure of treating the condition as well as resistance [13]. Furthermore, the patient–doctor relationship also causes the unnecessary prescribing by a general practitioner. A survey conducted in United Kingdom, which involved 1000 general practitioners, revealed that 55% of them had prescribed antibiotics, because they had felt under pressure [14]. Patients’ expectations are also an important factor for inappropriate antibiotic prescribing [15]. This could lead to the rise of antibiotic misprescribing and/or overprescribing, further impacting the development of resistance. Thus, it is important to address one’s knowledge and beliefs of the proper antibiotic usage, hence stopping the misconceptions and expectations of using antibiotics to treat minor illnesses [16,17].

According to the national statistics, the total population in Brunei Darussalam as of 2019 was 459,500 [18]. Brunei has four public hospitals, one for each of the four districts: Brunei/Muara, Tutong, Belait, and Temburong, with the Brunei/Muara district categorized as the urban area. Apart from hospitals, the Ministry of Health and the Ministry of Defense also provide fourteen health centers and eight medical clinics, respectively. The private health services comprise two hospitals, one health center, and 33 health clinics [19]. The healthcare access and quality index of Brunei was 76.4 in 2016, which was higher than that of the neighboring Malaysia (68.1) and Indonesia (44.5), but lower than that of Singapore (90.6) [20]. There are sixteen physicians per 10,000 population in Brunei [19], which is slightly higher when compared to the neighboring Malaysia (11 physicians per 10,000 population) [21].

The total antibiotic consumption in Brunei is 5.92 defined daily doses (DDD) per 1000 inhabitants per day, but this value is low due to the incomplete coverage and it only represents the use in public health [22]. The most commonly utilized were beta-lactam antibiotics, which represent 70% of the total consumption [22]. Under the Brunei Medicines Order 2007, all antimicrobials are categorized as prescription-only medicines, which means they can only be prescribed by registered medical practitioners, dentists, or veterinarians [23]. All products containing antibiotics, including eye drops, creams, and oral dosage forms, require a prescription for dispensing by a pharmacist [23]. Although antibiotics are obtained only by prescription, educating the public on the proper usage of antibiotics is still vital to ensure successful treatment and as a prevention measure against the spread of resistance. Hence, the Good Antibiotic Prescribing Practice (GAPP) is employed as one of the approaches in the antibiotic stewardship program. Brunei implemented a guidance document for the national antibiotic prescribing practice which was made effective in 2019. Brunei also joined the national action plan which is listed in the Ministry of Health 2019–2023 strategic plan on antimicrobial resistance that aligns with the World Health Organization Global Action Plan (WHOGAP) [24]. The five-year strategic plan will ensure that the antimicrobial resistance is decreased while ensuring effective treatment and prevention are in place [24]. The Brunei AMR Action Plan consists of four strategic objectives which include (i) awareness and education, (ii) surveillance and research, (iii) infection prevention and control, and (iv) optimized use of antimicrobials. These objectives are in line with the WHOGAP and the “One Health” approach to antimicrobial resistance.

In addition, the WHOGAP on antimicrobial resistance was implemented in 2015, the main goals whereof are to ensure antimicrobial agents are used rationally and to prevent infectious diseases with the proper use of effective and safe medicines of guaranteed quality accessible to all who are in need [22].

Multiple studies have been conducted regarding the knowledge of antibiotics and antimicrobial resistance among university students in other countries [25,26,27,28,29,30,31,32,33]. A study among undergraduate students in Sri Lanka revealed that senior students had good knowledge of antibiotics and antimicrobial resistance, although some had misconceptions about antibiotic use for viral illnesses [26]. A similar study in Malaysia also reported students had average knowledge in relation to antibiotics and good awareness of antibiotic resistance, although the study found that some respondents actually believed that antibiotics were useful in viral conditions [26]. This showed that despite having good knowledge of antibiotics and being aware of the essential information about antimicrobial resistance, the misunderstanding on the use of antibiotics in conditions related to viral infections was evident among undergraduate students.

To the best knowledge of the author, there are no documented Bruneian studies assessing the knowledge and perception of antibiotics. The Ministry of Health of Brunei often relies on the data of the neighboring countries as a yardstick in planning the public health campaign. Thus, this study is aimed to explore antibiotic usage and knowledge regarding antibiotics and antimicrobial resistance (AMR) among the Bruneian population, targeting specifically undergraduate students. This study could provide baseline data for future interventions and public health campaigning on antibiotic usage and antimicrobial resistance.

## 2. Materials and Methods

### 2.1. Study Design

This was a cross-sectional online survey study conducted from March to April 2020. The study involved undergraduate students from the Universiti Brunei Darussalam. The criteria of eligibility for the study were as follows: (i) adult (over 18 years old) and (ii) able to read and understand the Malay or English language. The students who were not willing to participate were excluded from the study.

An e-mail invitation was initially mailed to the Academic Registrar at the Institute of Health Sciences, then it was forwarded to all the Registrars from various faculties at the Universiti Brunei Darussalam. The data were collected from an online survey questionnaire. E-mail invitations for participation containing the online survey link and the information sheet for participants were disseminated to the targeted participants. To increase the response rate, reminder e-mails were forwarded twice to the contact persons following a two weeks interval. Other than reminder emails, the survey was also distributed among the students through WhatsApp application as a reminder.

### 2.2. Sample Size Calculation

Raosoft website was used to calculate the sample size with a confidence level set to 95% and the 5% margin of error [34]. Assuming a minimum population of 244, a sample of 150 participants was sought. The number required was calculated based on the proportional ratio.

### 2.3. Study Instrument

The questionnaire was adapted from the questionnaire based on the World Health Organization’s (WHO) “Antibiotic resistance: Multi-country public awareness” survey [35]. This questionnaire was preferred for the study, because it had been used previously by the WHO and contains relevant topics which cover the use of antibiotics and knowledge of antibiotics and antimicrobial resistance. The permission for reprinting and reproducing the survey was acquired from the WHO.

The online questionnaire was displayed in English, which is the medium of instruction at the university, and the questions were mostly close-ended. The questionnaire consisted of the following 5 sections: A: basic demographic data of the participants, including age, gender, nationality, years of study, and faculty; B: antibiotic use: 3 questions on the prior use of antibiotics, advice received, and the place of obtaining them; C: knowledge of antibiotics: 4 questions about the treatment duration, knowledge of sharing of antibiotics, disease conditions which require antibiotics; D: knowledge of antibiotic resistance: 5 questions on the commonly used terms related to AMR; 8 true/false statements regarding knowledge of AMR; 8 statements related to AMR measured using a five-point “Likert scale” of agreement, where 1 denotes “Disagree strongly”, 2 stands for “Disagree slightly”, 3—for “Neither agree nor disagree”, 4—for “Agree slightly”, and 5—for “Agree strongly”; E: antibiotic use in the community: one question on antibiotic use in agriculture and food products. The questions from the section on the knowledge of antibiotics and the true/false statements from the “Knowledge of antibiotic resistance” section were scored as follows: 1 for each correct answer and 0 for an incorrect one or “Do not know”. The knowledge scores for the questionnaire comprised of 12 questions with 14 correct answers were then categorized into “Good” (≥10 correct answers), “Moderate” (6–9 correct answers), and “Poor” (<6 correct answers).

### 2.4. Pilot Test

The questionnaire underwent preliminary pilot testing with subsequent revisions conducted among 15 respondents for initial validation. Necessary adjustments were made based on the feedback received from the respondents before finalizing the questionnaire. Three questions about obtaining antibiotics from a physician or nurse, vaccination, and washing hands from the original World Health Organization’s (WHO) “Antibiotic resistance: Multi-country public awareness” survey were omitted, as most antibiotics can only be obtained from a medical practitioner and the latter were found to be out of the research topic. The face validity of the online questionnaire was also assessed for readability, length, and relevance of the questions by two pharmacists with postgraduate qualifications at the Institute of Health Sciences, UBD.

The internal consistency of the survey questions was determined using the Cronbach’s alpha coefficient. The Cronbach’s alpha values of the section on antibiotic use and knowledge were 0.66 and 0.86, respectively, i.e., satisfactory.

### 2.5. Data Analysis

The data collected in the web-based survey’s database were exported into Microsoft Excel or other appropriate software. Data analysis was done using R statistical software (version 3.6) and Microsoft Excel. The data were summarized using descriptive statistics, which includes percentages, frequencies, and also means. Appropriate inferential statistics was also used according to the data distribution.

### 2.6. Ethical Approval

The study received ethical approval from the Research Ethics Committee of Pengiran Anak Puteri Rashidah Sa’adatul Bolkiah Institute of Health Sciences, Universiti Brunei Darussalam. All the participants were notified about the aims of the study, all the data collected were to remain anonymous, and confidentiality was strictly maintained. A written consent form was provided in the survey by ticking an option placed at the end of the cover letter prior to the distribution of the questionnaire. The study was conducted on a voluntary basis and respondents had the freedom to not participate in the online survey.

## 3. Results

### 3.1. Demographic Data

A total of 130 respondents returned a completed questionnaire yielding a response rate of 53%. The median age of the respondents was 21 years (SD = 2.2, range between 18 and 29). The majority of the respondents were female (76%, n = 99) and Bruneian (99%, n = 129). The proportions of the students in relation to the year of study (year 1–year 4) were 26% (n = 34), 18% (n = 23), 28% (n = 36), and 28% (n = 37). The students were mostly from the Faculties of Science (35%, n = 45), Health Sciences (50%, n = 65), Arts and Social Sciences (9%, n = 12), and from the School of Business and Economics (6%, n = 8). The characteristics of the respondents are presented in Table 1.

### 3.2. Antibiotic Usage

The results obtained for antibiotic use (Table 2) showed that 69% (n = 90) of the students reported having previously used antibiotics. More than half of the students obtained directions to take antibiotics from a healthcare professional (81%, n = 98) and 12% (n = 14) did not get advice. Antibiotics were mostly acquired from public (58%, n = 70) and private hospitals (31%, n = 37) or health clinics, as they can only be prescribed by a physician. However, few of the students reported getting antibiotics from a friend or family member (4%, n = 5) and 3% (n = 4) of them had the antibiotics saved up from the previous use.

### 3.3. Knowledge about Antibiotics and Antimicrobial Resistance

More than half of the students (51%, n = 66) showed a good level of knowledge (score more than 10) on antibiotics and antimicrobial resistance with a mean total knowledge score of 9 out of 14. Only 14% (n = 18) of the students scored below 6. Fisher exact test revealed that level of knowledge of the students was significantly different in relation to age (*p* = 0.001) and faculty (*p* < 0.001) but not between gender, nationality, place of resident, and year of study (Table 3). Good knowledge was found in students aged between 18 and 21 years old (*p* = 0.001) and in Health Sciences faculty (*p* < 0.001).

Almost all the students, 92% (n = 119), agreed that antibiotics should be taken as a full course as directed. The frequency and the percentage of students for the statements regarding antibiotic use are presented in Table 4.

**Figure 1:**
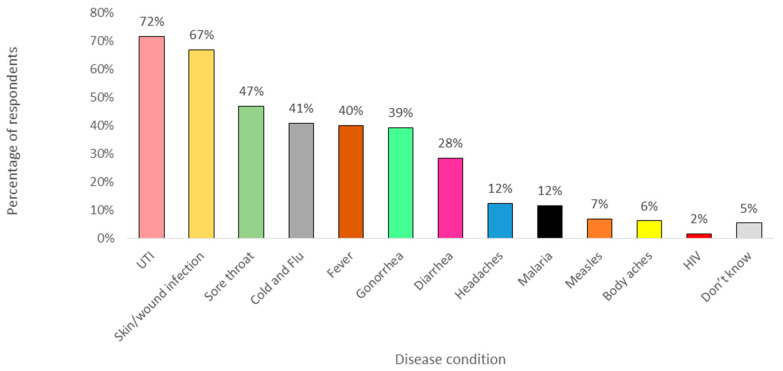
Percentages of the respondents who identified appropriate use of antibiotics in the various disease conditions. HIV: human immunodeficiency virus, UTI: urinary tract infection.

A large number of students correctly identified that antibiotics are used to treat bladder or urinary infections (UTI) (72%, n = 93), skin or wound infections (67%, n = 87), and gonorrhea (39%, n = 51) (Figure 1) [35]. Moreover, students incorrectly classified cold and flu (41%, n = 53) and fever (40%, n = 52) as conditions that can be treated with antibiotics. Similarly, the use of antibiotics was also reported to be appropriate for sore throat (47%, n = 61) and diarrhea (28%, n = 37).

**Figure 2:**
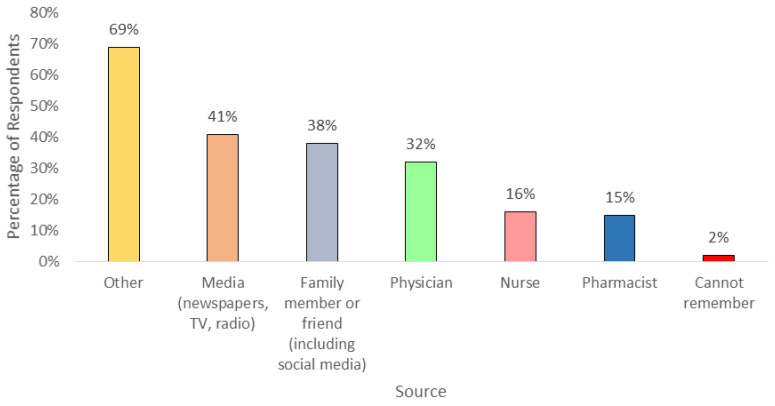
Percentages of respondent-reported sources of terminology.

When asked about the terminology, the students recognized the terms related to antibiotic resistance (89%, n = 116), drug resistance (78%, n = 102), antibiotic-resistant bacteria (72%, n = 94), and antimicrobial resistance (52%, n = 67). However, the acronym AMR (25%, n = 33) which stands for “antimicrobial resistance” and superbugs (25%, n = 33) were not well-known among the students. The most common source of the terminology was media (41%, n = 53), followed by a family member or friend, including social media (38%, n = 49), physician (32%, n = 41), nurse (16%, n = 21), pharmacist (15%, n = 19); other sources include pre-university and university lectures and secondary schools (69%, n = 90) (Figure 2).

In terms of knowledge about antimicrobial resistance (Table 5), 76% (n = 99) of the students mistakenly considered that antibiotic resistance occurs when the body becomes resistant to antibiotics. More than half of the respondents (83%, n = 108) agreed that when bacteria are resistant to antibiotics, treatment of infections can be very difficult or impossible. Some disagreed (52%, n = 68) with the statement “antibiotic resistance is only a problem for people who take antibiotics regularly”, but 17% (n = 22) of the respondents were not sure about it.

### 3.4. Knowledge about Antibiotic Use in Agriculture and Food-Producing Animals

Only few of the students (25%, n = 33) reported that they were aware about the use of antibiotics in agriculture and food products in the country, and more than half (69%, n = 90) of them were unsure about it.

## 4. Discussion

This study assessed the knowledge of antibiotics and antimicrobial resistance of the students. To the extent of the author’s knowledge, this is the first study carried out among Bruneian university students.

A quite high degree of antibiotic consumption was evident in the study. Sources of antibiotics were mostly public and private hospitals or health clinics, as all antimicrobials in Brunei are classified as prescription-only medicines. This is unlike many developing countries where antibiotics can be acquired without a valid prescription, which is commonly practiced among university students in China, India, and Nigeria [23,36,37]. The greater antibiotic consumption could be due to the easy access of healthcare in Brunei, which may cause excessive prescribing of antibiotics, and this correlates with a study in Malaysia where the rates of antibiotic prescribing were indeed high in both public and private primary care settings [38]. Thus, the first National Antibiotic Prescribing guidance published in 2019 was expected to minimize the unnecessary prescribing of antibiotics and to be adopted as a standard practice among all healthcare providers. This in part shares principles of the WHO’s systematic approach to the good prescribing practice in general, which can help minimize poor-quality and incorrect prescribing [39]. This can also be used as a referral for the prescribers to practice and ensure appropriateness of antimicrobial use. Further, Brunei also initiated a national strategic plan for 2019–2023 to tackle antimicrobial resistance follow the World Health Organization (WHO). This was implemented to ensure appropriate education and awareness is provided to the public and reduce the growth of resistance in the country.

Moreover, there were only few respondents found who had not received advice on how to take antibiotics and the need to complete the full course. Those who did not obtain advice are more likely to stop taking antibiotics when they feel better, which eventually leads to the growth of resistance in the community. Respondents also reported obtaining antibiotics from the previous use, but the rate was lower than in the previous studies in Malaysia (46/204), Qatar (161/596), China (1,965/11,192), and Saudi Arabia (165/347) [25,26,27,28]. There were more female students than male in this study, which is comparable to many other studies [8,25,29]. Similarly, a few respondents shared antibiotics with their friends or family members, which was also reported in Nigeria (5/400), the UK (3/242), and Jordan (76/1,158) [30,31,37]. The use of leftover antibiotics and antibiotic sharing by the respondents portrayed the non-compliance with antibiotic therapy and showed that the knowledge on antibiotic use is still lacking. Hence, it is important for healthcare providers to highlight the importance of taking antibiotics for the full course and advise patients not to stop taking antibiotics when the condition is better. Healthcare professionals can also recommend the public to return leftover antibiotics to prevent future use, which is unsafe and hence should be hindered. A pharmacist, too, can play a role in counselling patients on the possible risk if the course of antibiotic therapy is incomplete.

Furthermore, most of the students demonstrated good knowledge of antibiotics and antimicrobial resistance. The majority correctly identified conditions that can be treated with antibiotics, namely, bladder or urinary infections, skin or wound infections, and gonorrhea as suggested by the WHO in the antibiotic resistance survey. However, the misconceptions regarding the use of antibiotics for viral infections were also noted in the study, which was consistent with findings from other studies. The proportion was lower than in Jordan (527/1158), and Jatinangor, Indonesia (145/250), but higher than in Italy (210/1050) [31,32,33]. Sore throat and diarrhea were also reported appropriate for antibiotic use. Sore throat is a self-limiting viral illness which does not require the use of antibiotics and can usually be managed easily [40]. Lack of understanding on the difference between bacterial and viral infections could be the reason for the choices and this inappropriate choice can cause development of resistance if the erroneous belief is not addressed. In addition, respondents were aware of the terminology used related to antimicrobial resistance, but not the acronym AMR and superbugs. The reason for this is not known, but most likely because the acronym is rarely used to describe antibiotic or antimicrobial resistance in Brunei and the word “bug” is also not commonly used to identify germs, bacteria, or viruses. Pre-university and university lectures were the main source where the students heard about the term. There was a difference in the students from science and non-science faculties in recognizing terminology, since most of the respondents were from science faculties, namely, the Faculty of Science and the Institute of Health Sciences, so they were more exposed to the terminology than the non-science students. As for the knowledge of antimicrobial resistance, more than half of the respondents mistakenly believed that antibiotic resistance occurs due to the body becoming resistant to antibiotics, when in fact these are the bacteria that are resistant to antibiotics, and this was also seen in a study in Sri Lanka [41].

This study has several limitations. The results of the study may not be extrapolated to other universities, since it was conducted at one university. Other limitations of the study include the risk of response bias, as the findings of the survey were solely based on the self-reported data.

## 5. Conclusions

Although a good level of knowledge was found in this study, there are still gaps in areas of antibiotic use, especially the part on the appropriate antibiotic use for certain disease conditions, as some mistakenly considered antibiotic therapy for viral conditions. The current study findings provide baseline data for future research studies. However, further research is needed on this topic, especially to assess the attitudes and practice of antibiotic use among the students and not just focus on knowledge so that appropriate interventions can be carried out. Awareness of the proper use of antibiotics among the students is greatly required to correct their misconceptions and prevent the rise of antimicrobial resistance.

## Figures and Tables

**Table 1:** Demographic data of study respondents.

Demographic Data		Frequency (n)	Percentage (%)
**Gender**	Male	31	24
	Female	99	76
**Age**	18–21	68	52
	22–25	54	42
	26–29	8	6
**Nationality**	Bruneian	129	99
	International	1	1
**Place of residence**	Urban	103	80
	Suburban	26	20
**Year of Study**	Year 1	34	26
	Year 2	23	18
	Year 3	36	28
	Year 4	37	28
**Faculty**	Pengiran Anak Puteri Rashidah Sa’adatul Bolkiah Institute of Health Sciences (IHS)	65	50
	Faculty of Science (FOS)	45	35
	Faculty of Arts and Social Sciences (FASS)	12	9
	School of Business and Economics (SBE)	8	6

**Table 2:** Experience of study participants in antibiotic usage.

Statements	Frequency (n)	Percentage (%)
**When did you last take antibiotics?**		
In the last month	23	18
In the last 6 months	23	18
In the last year	16	12
More than a year ago	28	22
Never	9	7
Can’t remember	31	24
**Did you get advice from a physician, nurse, or pharmacist on how to take them?**		
Yes	98	81
No	14	12
Can’t remember	9	7
**On that occasion, where did you get the antibiotics?**		
Public hospital/health clinic	70	58
Private hospital/clinic	37	31
Pharmacy	3	2
The Internet	0	0
Friend or family member	5	4
I had them saved up from a previous time	4	3
Somewhere/someone else	0	0
Can’t remember	2	2

**Table 3:** Association of demographic characteristics with the level of knowledge. * Statistically significant *p* value.

Characteristics		Knowledge Score			*p* Value (Fisher’s Exact Test)
		Good	Moderate	Poor	
		n (%)	n (%)	n (%)	
**Gender**	Male	16 (24)	9 (20)	6 (33)	0.511
	Female	50 (76)	37 (80)	12 (67)	
**Age**	18–21	45 (68)	19 (41)	4 (22)	0.001 *
	22–25	18 (27)	23 (50)	13 (72)	
	26–29	3 (5)	4 (9)	1 (6)	
**Nationality**	Bruneian	65 (98)	46 (100)	18 (100)	1.000
	International	1 (2)	0	0	
**Place of Residence**	Urban	51 (78)	35 (76)	17 (94)	0.241
	Suburban	14 (22)	11 (24)	1 (6)	
**Year of Study**	Year 1	21 (32)	11 (24)	2 (11)	0.093
	Year 2	16 (24)	6 (13)	1 (6)	
	Year 3	15 (23)	13 (28)	8 (44)	
	Year 4	14 (21)	16 (35)	7 (39)	
**Faculty**	Pengiran Anak Puteri Rashidah Sa’adatul Bolkiah Institute of Health Sciences (IHS)	50 (76)	15 (33)	0	<0.001 *
	Faculty of Science (FOS)	14 (21)	22 (48)	9 (50)	
	Faculty of Arts and Social Sciences (FASS)	2 (3)	6 (13)	4 (22)	
	School of Business and Economics (SBE)	0	3 (7)	5 (28)	

**Table 4:** Knowledge of study participants about antibiotics.

Question/Statements	Frequency (n)	Percentage (%)
**When do you think you should stop taking antibiotics once you have begun treatment**		
When you feel better	10	8
When you have taken all of the antibiotics as directed	119	92
Do not know	1	1
**It is okay to use antibiotics that were given to a friend or family member as long as they were used to treat the same illness**		
True	6	5
False	117	90
Do not know	7	5
**It is okay to buy the same antibiotics or request them from a physician if you are sick and they helped you get better when you had the same symptoms before**		
True	26	20
False	91	70
Do not know	13	10

**Table 5:** Responses to various statements on antimicrobial resistance.

Statement	Correct Answer, n (%)	Incorrect Answer, n (%)	Unsure, n (%)
Antibiotic resistance occurs when your body becomes resistant to antibiotics and they no longer work as well	27 (21)	99 (76)	4 (3)
Many infections are becoming increasingly resistant to treatment by antibiotics	91 (70)	10 (8)	29 (22)
If bacteria are resistant to antibiotics, it can be very difficult or impossible to treat the infections they cause	108 (83)	9 (7)	13 (10)
Antibiotic resistance is an issue that could affect me or my family	97 (75)	14 (11)	19 (15)
Antibiotic resistance is an issue in other countries, but not here	87 (67)	9 (7)	34 (26)
Antibiotic resistance is only a problem for people who take antibiotics regularly	68 (52)	40 (31)	22 (17)
Bacteria which are resistant to antibiotics can be spread from person to person	52 (40)	39 (30)	39 (30)
Antibiotic-resistant infections could make medical procedures like surgery, organ transplantation, and cancer treatment much more dangerous	86 (66)	6 (5)	38 (29)
